# Explainable Boosting Machines Identify Key Metabolomic Biomarkers in Rheumatoid Arthritis

**DOI:** 10.3390/medicina61050833

**Published:** 2025-04-30

**Authors:** Fatma Hilal Yagin, Cemil Colak, Abdulmohsen Algarni, Ali Algarni, Fahaid Al-Hashem, Luca Paolo Ardigò

**Affiliations:** 1 Department of Biostatistics, Faculty of Medicine, Malatya Turgut Ozal University, 44210 Malatya, Turkey; 2 Department of Biostatistics, and Medical Informatics, Faculty of Medicine, Inonu University, 44280 Malatya, Turkey; 3 Department of Computer Science, King Khalid University, Abha 61421, Saudi Arabia; 4 Department of Informatics and Computer Systems, College of Computer Science, King Khalid University, Abha 61421, Saudi Arabia; 5 Department of Physiology, College of Medicine, King Khalid University, Abha 61421, Saudi Arabia; 6 Department of Teacher Education, NLA University College, Linstows Gate 3, 0166 Oslo, Norway

**Keywords:** rheumatoid arthritis, metabolomics, biomarker, machine learning, explainable artificial intelligence, explainable boosting machines

## Abstract

*Background and Objectives*: Rheumatoid arthritis (RA) is a chronic autoimmune disease characterised by joint inflammation and pain. Metabolomics approaches, which are high-throughput profiling of small molecule metabolites in plasma or serum in RA patients, have so far provided biomarker discovery in the literature for clinical subgroups, risk factors, and predictors of treatment response using classical statistical approaches or machine learning models. Despite these recent developments, an explainable artificial intelligence (XAI)-based methodology has not been used to identify RA metabolomic biomarkers and distinguish patients with RA. This study constructed a XAI-based EBM model using global plasma metabolomics profiling to identify metabolites predictive of RA patients and to develop a classification model that can distinguish RA patients from healthy controls. *Materials and Methods*: Global plasma metabolomics data were analysed from RA patients (49 samples) and healthy individuals (10 samples). SMOTE technique was used for class imbalance in data preprocessing. EBM, LightGBM, and AdaBoost algorithms were applied to generate a discriminatory model between RA and controls. Comprehensive performance metrics were calculated, and the interpretability of the optimal model was assessed using global and local feature descriptions. *Results*: A total of 59 samples were analysed, 49 from RA patients, and 10 from healthy subjects. The EBM generated better results than LightGBM and AdaBoost by attaining an AUC of 0.901 (95% CI: 0.847–0.955) with 87.8% sensitivity which helps prevent false negative early RA diagnosis. The primary biomarkers EBM-based XAI identified were *N*-acetyleucine, pyruvic acid, and glycerol-3-phosphate. EBM global explanation analysis indicated that elevated pyruvic acid levels were significantly correlated with RA, whereas *N*-acetyleucine exhibited a nonlinear relationship, implying possible protective effects at specific concentrations. *Conclusions*: This study underscores the promise of XAI and evidence-based medicine methodology in developing biomarkers for RA through metabolomics. The discovered metabolites offer significant insights into RA pathophysiology and may function as diagnostic biomarkers or therapeutic targets. Incorporating EBM methodologies integrated with XAI improves model transparency and increases the therapeutic applicability of predictive models for RA diagnosis/management. Furthermore, the transparent structure of the EBM model empowers clinicians to understand and verify the reasoning behind each prediction, thereby fostering trust in AI-assisted decision-making and facilitating the integration of metabolomic insights into routine clinical practice.

## 1. Introduction

Rheumatoid arthritis (RA) serves as a chronic autoimmune condition that produces persistent joint inflammation, which advances toward destructive joint damage, serious physical disability, and diminished life quality. RA affects 0.3% to 1% of the worldwide population and shows higher prevalence in women at 2–3 times compared to men across the global population [1,2]. The disease incidence rates grow significantly between ages 40 and 70. The global disease prevalence is most prominent among developed regions of North America and Northern Europe yet developing countries together with Asia experience increasing disease rates. Several pathophysiological determinants have been documented to explain the factors between genetic predisposition and environmental exposure to smoking and air pollution and life choices that lead to obesity and diet as contributors to RA development and disease progression. These increasing epidemiological burdens underline a greater need for innovative approaches to diagnosis and therapy to mitigate its global impact [3,4].

In such a complex and heterogeneous disease, RA represents one of the biggest challenges for early diagnosis, disease monitoring, and personalised treatment strategy; thus, it requires the identification of reliable biomarkers to guide clinical decisions. Clinical management of RA is further complicated due to its multifactorial nature involving genetic, environmental, and immunological factors. Recent metabolomics and artificial intelligence (AI) developments have emerged as transformative tools for understanding RA pathophysiology. Metabolomics allows for the detailed analysis of small molecules, giving insight into the metabolic pathways disturbed by disease activity. AI, more so explainable AI (XAI) methods, enhance the identification, analysis, and validation of biomarkers by integrating complex datasets and uncovering patterns that traditional biostatistics might miss. Together, these technologies can unravel metabolic changes and allow the construction of robust predictive tools that will provide a new avenue toward improved management of disease conditions and personalised therapies [5,6].

The advancement of metabolomics offers a complete investigation into small molecules in biological materials, providing information about metabolic pathway disturbances due to diseases. The strategy provides a method to find biomarkers that specifically identify diseases, including difficult cases like RA, despite diagnosis challenges. High-resolution techniques, like capillary electrophoresis coupled with quadrupole time-of-flight mass spectrometry, have been playing a pivotal role in untargeted metabolomics due to their precision, sensitivity, and possibility of detecting a wide range of metabolites in complex biological matrices. In light of this and by capitalising on the power of the advances mentioned above, the present study reanalysed publicly available RA patient metabolomics data, with a significant emphasis on identifying strong biomarkers that could clearly distinguish RA patients from healthy controls to enhance comprehension of the metabolic dysregulation inherently characteristic of RA [7,8].

Most biomarker discoveries in RA are grounded in an integrated approach involving biostatistics with advanced machine-learning models. Recent studies have emphasised integrating multi-omics data into machine learning for improved RA biomarker prediction accuracy. Recent approaches, such as XAI, in particular, include LIME, and more recent ones, like SHAP, are important for enhancing the interpretability of predictive models. These tools will help fulfil the urgent demand for transparent AI in healthcare so that model predictions can be comprehensible and trusted by clinicians [9,10,11]. The current study presents the results of a comprehensive workflow, to the best of our knowledge in the literature, that includes final metabolomics data preprocessing with univariate and multivariate approaches and model development based on machine learning concepts, and interpretable metabolomics biomarker discovery in RA with XAI-based EBM approach, to provide clinically relevant insights into RA pathogenesis and narrow the gap between computational analysis and practical clinical use. Post-hoc explanation tools like SHAP and LIME have brought progress to biomarker discovery [12,13], but they need to operate based on approximations of complex models which leads to possible inaccuracies between explanation results and actual model operations. EBM provides interpretability through a glass-box model structure, which builds interpretability directly into its operational architecture. The built-in transparency prevents approximation errors so model decision logic remains accurate when showing metabolite contributions including complex effects and mutual relationships between variables. EBM’s additive structure directly reveals the threshold concentrations of pyruvic acid that connect to RA risk levels whereas SHAP/LIME presents explanations through additive feature importance scoring. The EBM system achieves the best possible predictive AUC score of 0.901 through its combination of advanced predictive modelling and biologically significant findings thus creating a new paradigm for finding RA biomarkers, which address physician requirements for both accuracy and explanation.

The prediction and diagnosis of RA can be improved through machine learning algorithms among random forest and support vector machines with metabolomic data. The models use metabolomic profile patterns for high-powered biomarker identification purposes. A random forest algorithm enabled the development of a biomarker panel, which would accurately forecast RA through a synergy of machine learning with metabolomics in biomarker identification [14,15]. The predictive capabilities of boosting algorithms unite with interpretation methods in explainable boosting machines (EBM) to provide solutions beneficial for metabolomics biomarker research. The analysis through EBMs lets scientists identify how individual metabolite operations participate with each other when performing disease prediction, thus leading to more dependable diagnostic solutions. Additionally, the systematic implementation of evidence-based methods in RA metabolomics remains insufficient because researchers have not utilised them both for finding diagnostic biomarkers and analysing metabolite concentration thresholds. Such a research deficiency exists because EBMs could bridge the gap between clinical and computational aspects of RA management when they connect metabolomics patterns to actionable clinical decisions.

The current work further complements the growing literature on biomarkers in RA by integrating metabolomics with XAI in developing clinically applicable diagnostic tools. Based on the current state of the art of metabolomics and advanced AI methodologies, including frameworks of XAI, this research study presents a solid foundation for identifying and validating high-potential biomarkers for diagnosis and prognosis. Moreover, the integration of AI and metabolomics enhances not only the precision of RA diagnostics but also personalised treatment approaches. By underlining the transformative role of interdisciplinary approaches in RA research, this study points to new frontiers in managing autoimmune diseases that could have implications for other complex chronic conditions. In addition, the current paper undertakes these goals while creating an approach for combining XAI with rheumatology research as a method to discover biomarkers from complex autoimmune diseases through metabolomics-driven approaches.

## 2. Materials and Methods

### 2.1. Study Design, Dataset, and Sample Size

The current study used an existing experimental metabolomics dataset of RA patients and healthy controls. The participant recruitment process followed the strict requirements of the American College of Rheumatology (ACR). The current study gathered diagnostic blood samples from 50 patients diagnosed with RA by ensuring a standardised approach throughout the study group. This study eliminated one patient from analysis because renal haemodialysis influenced experimental outcomes while maintaining 49 patients diagnosed with RA for analysis. A blood test was performed on 10 healthy volunteers as controls because they matched RA patients for age and sex. The researchers performed this matching process to improve data validity by decreasing confounding variables. Inclusion criteria for RA patients were based on ACR classification criteria, including joint involvement, serologic markers, acute phase reactants, and symptom duration. Eligible participants were 18 years of age or older and were able to provide plasma samples. Exclusion criteria included other chronic diseases, renal failure requiring dialysis, active infections, and use of biologic therapy. Standardised blood sampling methods ensured all sample integrity throughout the study. The researchers immediately stored collected samples at 4 °C before processing them for metabolite degradation prevention for one hour. Ethylenediaminetetraacetic acid (EDTA) was used as an anticoagulant to separate plasma from whole blood because EDTA effectively keeps plasma metabolite conditions constant. The current study divided the plasma into smaller portions and kept them at −80 °C for biochemical composition maintenance before analysis. The analytical platform comprised of a combination between capillary electrophoresis-quadrupole time-of-flight mass spectrometry (CE-Q-TOFMS) for plasma metabolomic profiling.

CE-Q-TOFMS identifies polar metabolites essential for RA metabolic disruption assessment through precise metabolite analysis and quantification. The research delivered detailed qualitative and quantitative insights about RA-associated metabolomic changes using CE-Q-TOFMS technology [16]. The MetSizeR [17] program utilises probabilistic principal components analysis (PPCA) to calculate the necessary sample size through its power analysis method. The required participant sample size was determined through analysis at 0.05 false discovery rate (FDR) to be 18 participants distributed equally between both groups. Although MetSizeR estimated a minimum sample size of 18 participants (9 per group) at a 0.05 FDR threshold, we included 49 RA patients and 10 healthy controls to improve statistical power, capture greater biological variability, and enhance the generalisability of machine learning-based biomarker findings. Advantageous analyses were implemented using methods from three research fields that combined biostatistical approaches with advanced metabolomics procedures and XAI frameworks. Metabolite concentration data underwent multiple analysis methods, allowing an extensive assessment of metabolic shifts between groups. The integrated method sought to reveal important biological patterns while making difficult data assessments easier while searching for relevant clinical biomarkers.

### 2.2. Data Preprocessing

Class imbalance handling with SMOTE: To address class imbalance (49 RA cases vs. 10 controls), we applied the synthetic minority over-sampling technique (SMOTE) to the training set after splitting the dataset into training and test sets (4:1 ratio). SMOTE was implemented using the imbalanced-learn Python library (v0.10.1) with the following parameters [18]:

Sampling strategy: Set to balance classes at a 1:1 ratio (target ratio = auto).

k-neighbours: 5 nearest neighbours for synthetic sample generation.

Missing Value Imputation: Missing metabolite values were imputed using Multiple Imputation by Chained Equations (MICE) as the base estimator [19]. The process included:

Number of imputations: 10 iterations to capture variability.

Max iterations per imputation: 50 cycles, with early stopping if convergence criteria (tolerance = 0.01) were met.

Aggregation: Imputed values were averaged across iterations.

LightGBM hyperparameters for MICE included: n_estimators = 100, learning_rate = 0.1, max_depth = 3, and random_state = 42.

### 2.3. Biostatistical Data Analysis

The Kolmogorov-Smirnov test determined whether RA patients and control subjects would follow normal distribution patterns in their metabolite levels. We analysed demographic and clinical quantitative variables using the Student’s *t*-testing method, whereas qualitative variables were analysed using Fisher’s exact test. The analysis used standard deviation and mean for demographics, while frequencies served for qualitative variables. The Mann–Whitney U test was applied to analyse differences between groups because the metabolite levels did not show normal distribution. When significant differences were found, Cohen’s d effect size calculation was performed. The interpretation of effect size used Cohen’s d, where the small effect ranged from 0.20 to 0.50, and medium effect ranged from 0.50 to 0.80, and the large effect exceeded 0.80. Statistics showed significance at *p* < 0.05 as the cutoff for statistical significance. The research team applied the American Psychological Association (APA) 7.0 style to present all reported statistical differences. The mathematical calculations ran through IBM SPSS Statistics for Windows version 28.0, which operates from New York, NY, USA.

### 2.4. Machine Learning Algorithms and Performance Evaluation

In the study, classification models were created using AdaBoost, LightGBM, and EBM algorithms to distinguish RA and controls. AdaBoost was created by Freund and Schapire [20] to combine several algorithms into a strong, single model. This method combines the output classes from several models using a training dataset to build various models. Renowned as an ensemble learning method, AdaBoost combines separate models and dynamically reweighs to improve classification accuracy. The approach averages negative and positive samples for every feature to establish the weak classifier decision thresholds. AdaBoost then chooses the least error-prone weak classifiers for additional improvement and turns them into stronger classifiers, excluding the weak classifier attributes not included in the strong classifier. AdaBoost also creates a set of hypotheses, concentrating later hypotheses on cases that are ever more challenging to classify. The weighted majority vote of the classes projected by all hypotheses guides the ultimate choice. AdaBoost is a useful tool for raising the accuracy of classification techniques since it uses a methodical approach [21,22,23,24].

Particularly among decision tree methods, LightGBM—created by Microsoft in 2016—stands out in machine learning. Its rapid model training speed is particularly noteworthy, mostly because of its creative leaf-wise data training growth approach. Unlike previous gradient boosting systems, this method departs from the conventional depth-wise or level-wise techniques. LightGBM effectively lowers data volume by adopting the Gradient one-way sampling method, concentrating on pertinent dataset parts instead of the whole data pool. LightGBM has several benefits above other boosting techniques. These comprise fast processing, handling of vast data volumes, low RAM use, and improved forecast accuracy. It is also a flexible and economical choice since it enables GPU and parallel learning. LightGBM, an open-source system, expands on the successful gradient-boosting decision tree technology, highlighting Microsoft’s commitment to enhancing machine learning technologies [25,26,27,28].

EBM is a glass box model: a tree-based cyclic gradient-boosting generalised additive model (GAM) with automatic interaction detection. It excels in intelligibility and explainability and shows accuracy on par with cutting-edge machine-learning techniques, such as random forest and boosted trees. GAMs interpret the outcome as the sum of arbitrary functions for each characteristic, improving interpretability, unlike conventional models using simple weighted sums. With their small scale and fast forecasting powers, EBMs stand out because they can recognise and use special trait combinations—interactions. A group learning approach turns weak learners into strong ones, maximising performance. Leaf node count in EBM can be changed to suit additional performance optimisation. With a feature-wise boosting technique, the EBM boosting algorithm is painstakingly designed to concentrate on every feature independently during training cycles. This approach enables low learning rates, so the sequence of feature consideration is meaningless for the final model. Feature collinearity poses a major obstacle in model training since it could compromise interpretability and performance. EBM solves this using many rounds in the training phase, allowing exact identification of every feature’s contribution to the predicted output. EBM may also automatically identify and include pairwise interaction terms, improving predictive accuracy while preserving explainability. This function differs from conventional models that can depend on manual interaction term formulation, therefore complicating the model and hiding interpretability. By defining each feature’s unique influence on predictions, EBM’s additive character helps to explain it even more than more complicated, black-box models, which have sometimes opaque character. All things considered, EBM not only preserves the advantages of conventional GAMs but also advances them with increased accuracy, resilience, and in some situations, better explainability [29,30,31,32,33].

In the validation procedure of machine learning models, the data were initially divided into training and test sets at a ratio of 4:1, and in addition to the prediction results for the test set, 95% bootstrapped confidence intervals with 1000 repetitions were reported. Accuracy, F1-score, sensitivity, specificity, positive predictive value, negative predictive value, area under the curve (AUC), and the Brier score were calculated to evaluate the performance of the models in distinguishing RA and healthy controls. While accuracy indicates the overall correct classification rate of the model, F1-score was used as a criterion that balances sensitivity and specificity. The model’s correct identification of positive examples becomes measurable through sensitivity, whereas specificity measures its ability to spot negative examples correctly. The positive predictive value describes how often positive predictions prove accurate, whereas the negative predictive value shows the percentage of correct negative outcomes. The evaluation process of model discrimination power relied on calculating AUC, and the Brier score assessed the precise nature of the predictions and the model quality to determine calibration. An assessment involving Brier scores determined the well-calibrated model through the identification of the model with the lowest score [34,35,36].

### 2.5. Global and Local Explanations with Explainable Boosting Machine

Machine learning models require explainability features to enable the understandability of their decision processes, specifically when discovering metabolomic biomarkers. The transparency in these systems helps researchers analyse the vital metabolomic elements that affect model prediction and provides crucial knowledge about the algorithmic processes used during real-time applications. EBM implements a generalised additive model through tree-based architecture to provide feature ranking and visualise interacting variables. The interpretability system offers two levels of analysis to display model-wide behavioural understanding together with per-instance-specific explanations, which overcome the typical accuracy level versus explainability trade-off. EBM demonstrates an important capability to produce complete explanations by utilising only training data information. A completely transparent method helps people see and quantify how features affect predictions of RA assessment. Irrespective of traditional importance rankings, the application allows users to view the precise effects of features across different value ranges. The learning process of the algorithm becomes detectable by inspecting the pattern of feature coverage, which shows how it enhances its grasp of the relationship between features. The ability to see through the model confirms its decision making while assisting in biological discoveries linked to disease root causes [37,38,39].

## 3. Results

The study analysed metabolomic data from 59 participants (49 RA patients and 10 controls). The mean age of the participants in the study was 63 ± 14 years in the control group and 60 ± 13 years in the RA group, and the difference between the groups was not statistically significant (*p* = 0.540). Regarding gender distribution, there were 10 females in the control group and 43 females and 6 males in the RA group. There was no statistically significant difference between the groups regarding gender (*p* = 0.577). The mean DAS28-ESR score showing disease activity in RA patients was 3.71 ± 1.23, and the score varied between a minimum of 1.12 and a maximum of 7.62. Regarding treatment, 39 of the RA patients used methotrexate (MTX), and 22 of them used glucocorticoids (GC). Table 1 shows the changes in metabolite levels in the RA and control groups.

The dataset controls indicated that no issues could impact the data preprocessing and modelling phases. Initially, the data was divided into training and test sets in a 4:1 ratio, with all preprocessing operations conducted independently for each set. Missing values in the dataset were imputed using the LightGBM-supported MICE approach. The SMOTE technique was utilised to equilibrate the distribution of positive and negative classes, hence mitigating biased outcomes in machine learning prediction models. Subsequent analyses were conducted on the balanced dataset. Following the data pretreatment phase, the modelling phase commenced. The EBM model was trained without interaction terms, in addition LightGBM and AdaBoost models were trained for comparative performance analysis. The test classification performance measurements of the respective models are presented in Table 2 with 95% confidence. Table 2 reveals that all three models had an impressive sensitivity score in predicting RA. Heightened sensitivity results in a reduction in false negatives (FN). In comparative biological research, false positive and false negative errors frequently occur. Consequently, it is crucial to ascertain the chance that a genuine effect is considerable. A reduced FN value signifies a favourable outcome for RA samples. This outcome is critically important as the main objective of this study is to reduce missing rheumatoid arthritis cases (false negatives). In the comparison of model performances, the EBM model demonstrated superior robustness relative to the LightGBM and AdaBoost models. The EBM model attained a commendable discrimination outcome with an AUC value of 0.901. The study produced the Brier score, alongside detailed performance measures, to assess the calibration of the prediction models. A lower Brier score indicates superior model calibration, and upon analysis, the EBM model attained the lowest Brier value.

**Figure 1:**
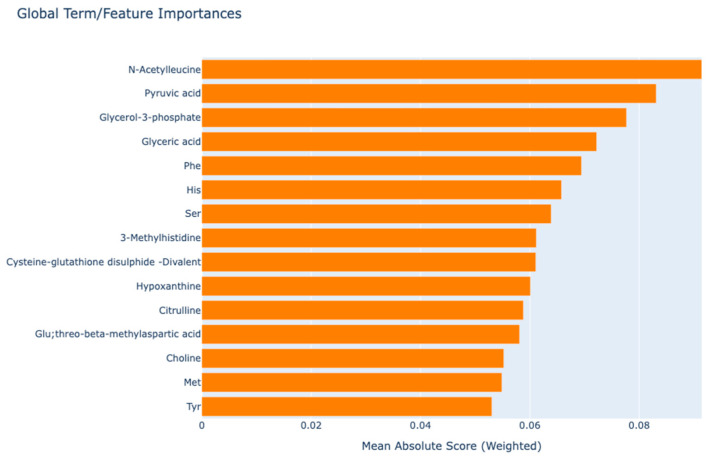
Global interpretation graph to explain model predictions.

The EBM algorithm is a generalised additive model derived from a tree-based model. Features can be prioritised and visualised to demonstrate their influence on individual predictions from both global and local perspectives based on their contributions. The comprehensive annotation of EBM facilitates the visualisation of each metabolite’s influence on the anticipated results of RA. Figure 1 presents a comprehensive summary of global annotations, highlighting the significance of all components. The feature importance scores shown in Figure 1 were calculated as the weighted average absolute values of the shape functions learned by the EBM model, reflecting each metabolite’s contribution to overall prediction performance. The weighted average absolute scores of metabolites importance for the EBM model are presented in the global annotation graph. The EBM model, without interaction terms, identified the most important principal biomarker candidates: “*N*-Acetyleucine”, “Pyruvic acid”, and “Glycerol-3-phosphate” (Figure 1).

**Figure 2:**
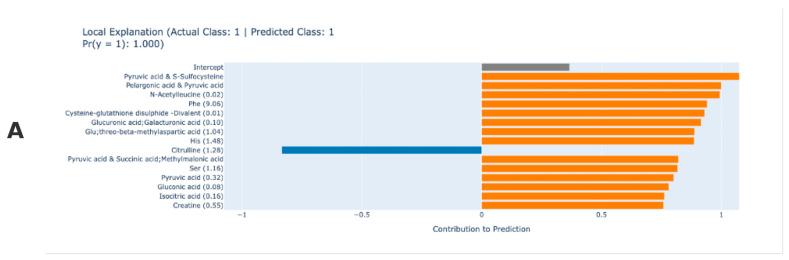

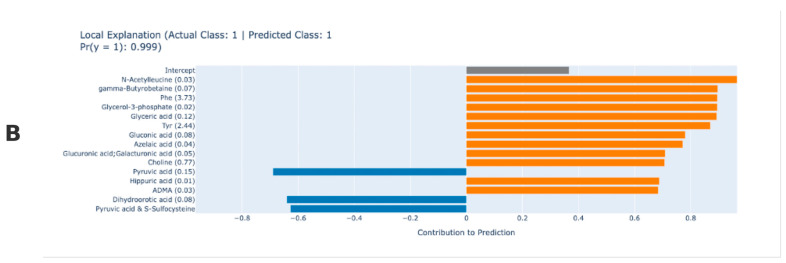
Explainable boosting machine local interpretation. (**A**) Local explanation for a correctly predicted sample. Orange bars indicate metabolites positively contributing to the prediction, while blue bars (e.g., Pyruvic acid & Succinic acid) show negative contributions. Key contributors include Citrulline and His. (**B**) Local explanation for another correctly predicted sample. Similar to (**A**), orange bars denote positive contributions (e.g., gamma-Butyrobetaine, Phe), and blue bars (e.g., Dihydroorotic acid) reflect negative influences. Notable differences in metabolite rankings highlight variability in feature importance across samples. Color key: Orange = Positive contribution; Blue = Negative contribution.

This EBM local explanation plot explains why the model assigned an individual to the RA class. The EBM algorithm allows to give detailed contributions of metabolites for a single prediction. As an example, Figure 2A,B show the results of a typical individual RA prediction. Based on the predicted results, the EBM model classified the patient in Figure 2A as RA with 100% probability and the patient in Figure 2B with 99.9% probability. The model classified the observation in Figure 2A as having RA with 100% probability, with some metabolites strongly supporting this classification for the current patient, while others have the opposite effect. Positive contributing variables are metabolites that support the model’s RA diagnosis and may be associated with the disease. Pyruvic acid and S-sulfocysteine, pelargonic acid and pyruvic acid, *N*-acetyleucine, phenylalanine (Phe), cysteine-glutathione disulphide—divalent, glucuronic acid, glu, threo-beta-methylaspartic acid, histidine (His), serine, pyruvic acid, gluconic acid, isocitric acid, and creatine are the compounds that contribute the most to the RA diagnosis according to the model prediction results for the relevant patient. Pyruvic acid, in particular, is associated with energy metabolism and may be linked to oxidative stress, while amino acids such as Phe and His may play a role in inflammatory processes. The patient’s citrulline value, which contributed negatively, had an opposite effect on the model’s RA prediction. EBM-based interpretability is crucial for RA identifying. It has superior predictive accuracy in attributing interpretability to output results (Figure 2).

**Figure 3:**
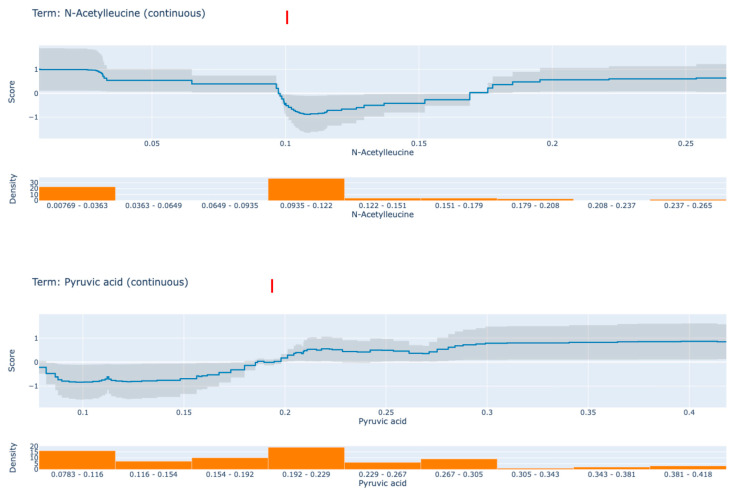
Contribution of *N*-acetyleucine and pyruvic acid metabolites to the distinction of rheumatoid arthritis. In the upper part of the graph, the scores of the model corresponding to different values of the metabolite are given, and in the lower part, the data density is given. Red dashed vertical lines mark key thresholds (≈0.1 and ≈0.2, respectively) where the model’s score shifts notably, providing enhanced interpretability regarding how these metabolites influence the predicted probability of disease.

EBM is highly comprehensible due to the quantification of the contribution of each feature to the ultimate prediction of an individual. Each shape function is a complex nonlinear function that is obtained through gradient boosting and bagging. The order of the features is irrelevant when the boosting procedure is limited to training circularly on one feature at a time with a very low learning rate. In order to mitigate the impact of collinearity, the model iterates through the features in order to determine the optimal feature shape function for each. It demonstrates the extent to which each feature contributes to the model’s prediction. Ultimately, the final prediction for each individual is determined by adding up all of the feature shape functions for the specified features. Figure 3 displays the contribution values of the two most significant biomarker candidate metabolites to the prediction results in accordance with the global EBM. The top line diagram displays the metabolites’ contribution for each subfigure, with the predicted accuracy as indicated by the grey band. The data density of the metabolite in question is represented by the bar graph below. The trend of the line graphs of individual metabolites can be analysed to determine the impact of each factor on RA. The following discussion pertains to two designated metabolites that have a greater influence on the anticipated outcomes of RA.

Figure 3 presents partial dependency plots for two key metabolites derived from the CPA model: *N*-acetyleucine (Panel A) and pyruvic acid (Panel B). These plots show how changes in metabolite levels affect the model’s predicted RA probability. In Panel A, *N*-acetyleucine shows a nonlinear relationship with the model score. At levels below approximately 0.1, the model score remains high, indicating an increased predicted risk for RA. However, when *N*-acetyleucine levels exceed 0.1, the model score drops sharply, indicating a decrease in the predicted RA probability. This trend suggests that lower *N*-acetyleucine levels may be associated with the presence of disease, whereas higher levels may play a protective role, implying a threshold-dependent effect. In Panel B, pyruvic acid shows the opposite pattern. At concentrations below 0.2, the model score remains low, indicating a lower RA risk. However, when pyruvic acid levels exceed 0.2, the model score increases rapidly, indicating a higher probability of RA. This model supports the hypothesis that high pyruvic acid levels are positively associated with RA, and therefore, pyruvic acid may serve as a candidate biomarker for disease detection. These findings highlight the capacity of EBM to reveal nonlinear and interpretable relationships between metabolite levels and disease risk, reinforcing the clinical relevance of threshold effects in metabolomic profiles. As a result, levels of pyruvic acid in RA patients may reflect enhanced glycolysis and oxidative stress associated with chronic inflammation, while abnormal *N*-acetyleucine patterns may indicate dysregulated amino acid metabolism with potential immunomodulatory effects, suggesting their relevance as functional biomarkers (Figure 3).

## 4. Discussion

The present research examined RA participant metabolomics levels alongside healthy subjects while constructing explainable machine learning methods to determine predictive biomarkers. Uniformed data integration with XAI techniques demonstrated how metabolic problems linked to RA became visible while establishing these methods for clinical diagnostic testing. The research examined 49 RA patients and 10 healthy subjects using samples which demonstrated no differences in participant age or sex characteristics for the prevention of demographic confounding errors. The disease activity score 28 (DAS28-ESR) assessed a moderate 3.71 ± 1.23 mean score on patients with rheumatoid arthritis, thus validating the need for therapeutic intervention. The study shows that observed metabolic changes in RA patients occurred despite their regular treatment with methotrexate (79.6%) and glucocorticoids (44.9%), which supports standard RA treatment protocols [40]. The univariate analysis detected 25 metabolites which presented significant statistical differences (*p* < 0.05) in patients with rheumatoid arthritis. The substantially elevated levels of both lactic acid (*p* = 0.004) combined with pyruvic acid (*p* < 0.001) demonstrate how cells undertake anaerobic metabolism as a characteristic feature of inflammatory processes. The inflammatory process associated with endothelium dysfunction likely controls phenylalanine elevation (*p* < 0.001) while decreasing citrulline levels (*p* = 0.002). RA synovium may experience exacerbated oxidative stress because of decreased carnitine (*p* = 0.008) along with choline (*p* = 0.007). The disease course of RA progresses as immune and vascular systems begin to affect each other. The impaired condition leads to fat acid molecule accumulation that increases tissue damage and inflammatory activity. Studies produce contradictory outcomes on metabolic reprogramming since the method establishes anti-inflammatory settings to show that metabolism influences immune responses through complicated pathways [41]. The significant reduction in cysteine-glutathione disulfide (*p* < 0.001) and its divalent form (*p* < 0.001) points to glutathione depletion, a critical antioxidant defence mechanism. A study supports previous findings about redox imbalance in rheumatoid arthritis pathogenesis. The levels of 3-methylhistidine increase in RA patients (*p* = 0.001), which might indicate the development of cachexia from RA [42].

The EBM generated better results than LightGBM and AdaBoost by attaining an AUC of 0.901 (95% CI: 0.847–0.955) with 87.8% sensitivity which helps prevent false negative early RA diagnosis. A high sensitivity factor minimises incorrect diagnoses and remains essential since improper treatment delays result in permanent joint damage. The EBM has better calibration capabilities than alternative models because it presents the lowest Brier score of 0.129 which supports its reliability for clinical risk stratification [43]. EBMs delivered superior model performance compared to LightGBM and AdaBoost although both algorithms showed respectable outcomes because their lower sensitivity and elevated Brier scores indicated a relationship between prediction power and interpretability standards. The elevated sensitivity level of 0.878 makes the EBM model optimally suited for clinical usage where false negative errors produce serious implications. The model prevents incorrect negative diagnoses to reduce the number of patients who have undiagnosed rheumatoid arthritis since the disease progresses steadily after diagnosis. Symptoms make EBMs desirable when identifying biomarkers because this model enables detailed observations of both global and local feature contributions. The global interpretation graph showed *N*-acetyleucine together with pyruvic acid and glycerol-3-phosphate as the cardinal metabolites, which significantly predicted the development of RA. Medical practitioners need transparency to understand prediction rationale because this knowledge enables them to incorporate predictions into their professional practice [44]. The local interpretation plots delivered complete information about which individual metabolites functioned during RA classification. This research evaluated RA risk and pyruvic acid relation by confirming that lower concentrations generated safety yet elevated concentrations drastically increased RA diagnosis probability. Physical insights from these observations provide essential knowledge for the development of precise disease detection methods and specific therapy plans. Studies have verified that amino acids serve as important elements for RA risk assessment because they play a role in inflammatory pathways [45].

The global feature importance analysis of the EBM model revealed *N*-acetyleucine, pyruvic acid, and glycerol-3-phosphate as key contributors to the prediction of RA. In the partial dependence plots shown in Figure 3, both *N*-acetyleucine and pyruvic acid exhibited non-linear threshold-based effects, reflecting biologically plausible patterns relevant to RA pathophysiology. Specifically, pyruvic acid displayed a clear threshold effect: the EBM model’s score remained low at concentrations below approximately 0.2, suggesting low RA risk. However, once levels exceeded this threshold, the model score sharply increased, indicating a higher probability of RA diagnosis. This is consistent with previous studies reporting that pyruvate accumulation contributes to mitochondrial dysfunction in RA fibroblasts, suggesting that elevated pyruvic acid levels may serve as a metabolic indicator of inflammatory stress and energy imbalance. In contrast, *N*-acetyleucine exhibited a distinct non-linear pattern. At lower concentrations (below ~0.1), the model score remained high, indicating increased RA risk. Beyond this point, the model score declined, suggesting a protective effect at higher levels. This inverse threshold behaviour implies that *N*-acetyleucine may exert immunomodulatory or anti-inflammatory effects once a critical plasma level is surpassed. Contrary to prior interpretations suggesting a U-shaped relationship, the updated findings indicate that RA risk decreases continuously after crossing the 0.1 threshold, with no secondary increase in model score at high concentrations. This pattern supports the idea that *N*-acetyleucine may be a context-dependent metabolite, where its biological function transitions from potentially deleterious to protective depending on concentration. Glycerol-3-phosphate from glycolysis served as a risk factor in developing RA because it positively affected RA development [46,47]. The biological importance of metabolites detected through the EBM model establishes their clinical significance. *N*-acetyleucine exhibited a threshold-dependent relationship with RA risk, where concentrations below approximately 0.1 were associated with increased risk, while higher concentrations demonstrated a protective effect, indicating a transition from risk-enhancing to risk-reducing behaviour. Research investigations on RA pathogenesis pathways should receive guidance from this discovery [48]. The link between pyruvic acid and energy metabolism and oxidative stress makes it suitable for use as a biomarker in RA disease progression assessment. Higher levels of pyruvic acid support its readiness as a diagnostic indicator by potentially indicating inflammatory conditions through increased glycolytic activities. The research analysis revealed citrulline functions as a compound that produces adverse effects toward RA diagnosis. The research data supports existing findings which demonstrate lowered citrulline levels occur in people with RA because their arginine metabolic processes function poorly. Such elaborate connections between biomarkers help demonstrate EBM model potential for discovering new diagnostic elements that improve rheumatoid arthritis understanding [49]. The local interpretability feature of the model generated individual-specific metabolic factors, including phenylalanine and histidine for custom dietary, as well as therapeutic intervention planning. The redox imbalance of particular patient groups can potentially be reduced through both interventions targeting amino acid transporters along with glutathione precursor supplementation. The negative predictive power of citrulline in the model supports its function as a nitric oxide synthesis agent which suggests endothelial repair occurs in specific RA patient subgroups [50].

*N*-acetyleucine demonstrated a threshold-dependent inverse association with RA risk. At concentrations below ~0.1, the model’s output remained elevated, indicating increased RA likelihood. However, after surpassing this threshold, the EBM score sharply declined, suggesting a protective effect at higher levels. Contrary to previous assumptions of a U-shaped relationship, our findings indicate a single inflection point beyond which increased concentrations are associated with reduced disease risk. This may reflect the metabolite’s role in immune regulation or anti-inflammatory activity, and it supports the need to revisit previous interpretations. Clinically, *N*-acetyleucine levels may inform early diagnosis and personalised treatment strategies, particularly as part of pre-treatment evaluations or metabolic monitoring protocols.

Pyruvic acid showed a non-linear but strongly positive effect in the EBM model. The model score remained low at concentrations below 0.2, while a sharp increase occurred above this threshold, pointing to an elevated RA risk. This result is consistent with studies linking pyruvate accumulation to mitochondrial dysfunction and altered glycolysis in RA fibroblasts, emphasising its potential as a diagnostic and prognostic biomarker [51]. Since both metabolites can be measured through non-invasive biological samples (e.g., plasma or serum), their use is advantageous for clinical laboratory integration. Moreover, threshold-based effects of these metabolites emphasise the need for concentration-aware therapeutic strategies. The ability to uncover such relationships using transparent models like EBM highlights their potential for clinical decision support systems, enabling clinicians to understand and trust model outputs. Our study affirms and extends findings from previous metabolic research into RA. While prior hypotheses suggested a U-shaped effect of *N*-acetyleucine [51], our results instead reveal a threshold-dependent monotonic decrease in risk, reinforcing the concept of hormetic or biphasic behaviour, where dose determines biological response. This pattern mirrors mechanisms observed in immunopharmacology and metabolism, where low concentrations activate, but high concentrations suppress, or vice versa [51].

Additional EBM-derived features, such as phenylalanine, histidine, and citrulline, also demonstrated predictive relevance and may support precision interventions. For example, *N*-acetylcysteine supplementation may restore redox balance and glutathione homeostasis in RA patients, mitigating oxidative damage associated with disease progression. Likewise, L-citrulline may act as a vascular repair agent in RA subgroups, given its role in nitric oxide synthesis, as suggested by preclinical studies [52,53].

EBM represents a glass-box structure because its architecture enables built-in model interpretability thus differentiating it from post-hoc explanation tools, such as SHAP and LIME. By removing misalignment between model output reactions and interpretation EBM delivers trustworthy feature influence analysis (e.g., metabolite effects) that matches with the decision reasoning process. EBM demonstrates an explicit additive structure that shows how pyruvic acid creates a non-linear link to RA risk, which matches its biological role in glycolysis and oxidative stress even though SHAP/LIME might explain these effects through additive approximations. EBM detects non-linear metabolite effects, including threshold-based behaviours such as that observed for *N*-acetyleucine, without any need to define them manually in the model, while simultaneously detecting pairwise interactions between features autonomously. SHAP and LIME implement an assumption of additive feature contributions involving metabolic pathways but this approach might simplify their analysis of such complex biological systems. The Brier score results demonstrate that EBM offers superior accuracy for risk assessment compared to LightGBM and AdaBoost where its calibration achieved 0.129 compared to their, respectively, higher scores of 0.146 and 0.187. SHAP/LIME provides explainability for models with high accuracy, but do not function as standalone solutions for enhanced performance. Our approach resolves this clinical requirement through predictive excellence (AUC = 0.901) in combination with actionable diagnosis information (such as metabolite thresholds) to meet both high performance needs and interpretation requirements. Our research showed that *N*-acetyleucine levels below approximately 0.1 were associated with an increased RA risk, whereas higher concentrations beyond this threshold exhibited a protective effect, which supports the metabolite’s known role in immunological regulation. This important detail would remain unidentifiable through most other XAI methodologies [37,54]. Beyond RA, osteoarthritis (OA)—another major arthritic condition—similarly imposes significant socioeconomic burdens due to its rising prevalence and limited disease-modifying therapies. Current OA interventions focus on symptom management (e.g., analgesics, physical therapy), yet emerging evidence highlights central pain sensitization as a shared therapeutic target across inflammatory and degenerative joint diseases [55]. The gut microbiota functions as an important modulator of musculoskeletal health because bioactive small molecules mediate the systemic effects on immune, metabolic, and endocrine pathways. Microbiota composition together with musculoskeletal outcomes may be influenced by genetic components combined with dietary elements and lifestyle variables including physical activity thus requiring individualised strategies in RA treatment. Competitive athletes show that exercise improves their prognosis but their research does not translate to non-competitors since they possess distinct microbiota profiles and metabolic needs. Multidimensional analysis of microbiome and metabolic markers such as *N*-acetyleucine and pyruvic acid has the potential to reveal new pathways between gut signals and rheumatoid arthritis development despite being in line with present-day demands for distributed omics methodologies in diagnostic medicine [56].

The study has some limitations. The cross-sectional approach may prevent researchers from drawing conclusions about possible cause-and-effect relationships between the observed metabolic effects. While the 10 participants in the control group may have reduced the generalisability of the study, the statistical analysis with 1000 replicates increased the accuracy of the parameter ranges. External validation using larger cohorts is needed to validate the findings and the predictive model derived from SMOTE addressing class imbalance issues. Although explanatory models such as EBM are more resistant to overfitting than black-box algorithms, the small sample size combined with high-dimensional metabolomics data still poses a risk of model overfitting. Furthermore, the lack of an independent external validation dataset limits the ability to assess the generalisability of the model to larger clinical populations. Future studies with larger and more diverse cohorts are needed to validate these findings. The lack of available data on serological markers, including anti-CCP, prevents immediate comparison with current diagnostic criteria [57]. The assessment of metabolite measurements before and after treatment through longitudinal research would establish their behaviour during disease progression and treatment reaction. It is necessary to conduct mechanistic laboratory tests that demonstrate the functional cellular effects of immune cell activation resulting from prioritised metabolites including *N*-acetyleucine. Combining multiple types of omics data like proteomics along with transcriptomics and clinical variables will improve both predictive power and biological explanation of the system. Assessment of pre- and post-treatment metabolite measurements through long-term studies will determine their behaviour during disease progression and treatment response. It is necessary to conduct mechanistic laboratory tests that demonstrate the functional cellular effects of immune cell activation resulting from priority metabolites, including *N*-acetyleucine. Combining multiple types of omics data, such as proteomics, with transcriptomics and clinical variables will improve both predictive power and biological explanation of the system.

## 5. Conclusions

The XAI-based EBM model developed in this study provides a reliable and interpretable structure that can be integrated into clinical decision support systems for RA diagnosis. The high sensitivity level of the model provides a significant advantage in clinical decision processes in terms of minimising the false negative classification of RA cases in the early period. This is of critical importance in terms of preventing late initiation of treatment due to misdiagnosis and preventing joint damage from progressing. When considering real-world applications, the model’s ability to provide both global and local explanations facilitates physicians’ decision making at both the population and individual patient level; and provides supportive information in many areas such as treatment planning, metabolic pathway targeting, and patient education. In particular, the fact that biomarkers such as pyruvic acid and *N*-acetyleucine are associated with RA-related metabolic disorders enables these molecules to be evaluated as diagnostic and potential therapeutic targets. This is an important result in terms of integration into personalised medicine approaches. The EBM framework combines reliable prediction capabilities with explainability functions to connect artificial intelligence technology to clinical care, thus laying the foundation for precision rheumatological treatment methods.

## Figures and Tables

**Table 1:** Univariate statistical analysis results.

Metabolite Name	Control	RA	*p*-Value	ES
	Median (Min–Max)	Median (Min–Max)		
1-Methyladenosine	0.01 (0.007–0.013)	0.009 (0.006–0.019)	0.269	
1-Methylnicotinamide	0.018 (0.008–0.036)	0.016 (0.009–0.068)	0.099	
2-Amino-2-(hydroxymethyl)-1,3-propanediol	0.181 (0.063–1.071)	0.088 (0.054–1.168)	<0.001	0.54
2-Aminoadipic acid	0.021 (0.014–0.03)	0.023 (0.014–0.049)	0.092	
2-Aminobutyric acid	0.369 (0.221–0.473)	0.355 (0.207–0.699)	0.823	
2-Hydroxybutyric acid; 2-Hydroxyisobutyric acid	0.228 (0.117–0.287)	0.246 (0.129–0.522)	0.032	0.60
2-Hydroxypentanoic acid	0.097 (0.059–0.165)	0.091 (0.055–0.344)	0.400	
2-Oxoglutaric acid	0.086 (0.067–0.108)	0.087 (0.057–0.123)	0.491	
2-Oxoisovaleric acid; 4-Oxovaleric acid	0.092 (0.073–0.122)	0.098 (0.06–0.15)	0.010	0.43
3-(2-Hydroxyphenyl)propionic acid; 3-Phenyllactic acid; Tropic acid;3-Ethoxybenzoic acid; 3-(4-Hydroxyphenyl)propionic acid	0.031 (0.024–0.062)	0.03 (0.016–0.087)	0.211	
3-Hydroxybutyric acid	0.244 (0.1–0.422)	0.157 (0.072–1.025)	0.006	0.01
3-Indoxylsulfuric acid	0.073 (0.032–0.183)	0.085 (0.017–0.659)	0.873	
**3-Methylhistidine**	**0.132 (0.081–0.375)**	**0.207 (0.062–0.81)**	**0.001**	**0.66**
3-Phenylpropionic acid	0.032 (0.028–0.046)	0.03 (0.023–0.085)	0.175	
4-Methyl-2-oxopentanoic acid; 3-Methyl-2-oxovaleric acid	0.552 (0.409–0.81)	0.537 (0.283–0.952)	0.851	
5-Oxoproline	0.174 (0.132–0.218)	0.16 (0.108–0.22)	0.016	0.51
ADMA	0.023 (0.02–0.029)	0.027 (0.016–0.038)	0.002	0.57
ADP;dGDP	0.017 (0.009–0.054)	0.012 (0.003–0.08)	0.154	
Ala	5.065 (2.838–7.465)	5.521 (3.686–11.994)	0.035	0.35
Allantoin	0.113 (0.088–0.138)	0.113 (0.064–0.158)	0.334	
Arg	2.676 (1.857–3.427)	2.556 (1.191–4.405)	0.318	
Asn	0.699 (0.61–0.975)	0.726 (0.481–1.234)	0.449	
Asp	0.064 (0.045–0.083)	0.064 (0.04–0.356)	0.672	
Azelaic acid	0.024 (0.02–0.035)	0.026 (0.01–0.058)	0.004	0.57
Benzoic acid	0.034 (0.027–0.047)	0.037 (0.024–0.077)	0.170	
beta-Ala	0.044 (0.027–0.067)	0.046 (0.026–0.175)	0.218	
Betaine	1.775 (1.066–2.106)	1.603 (0.922–2.975)	0.453	
Carnitine	2.812 (2.112–3.388)	2.531 (1.701–3.355)	0.008	0.61
Cholic acid	0.024 (0.009–0.183)	0.029 (0.006–0.183)	0.272	
**Choline**	**1.023 (0.715–1.317)**	**0.87 (0.535–2.133)**	**0.007**	**0.30**
Citric acid	1.692 (1.37–2.293)	1.6 (1.018–2.827)	0.520	
**Citrulline**	**0.823 (0.626–1.246)**	**0.676 (0.196–1.826)**	**0.002**	**0.46**
Creatine	1.647 (0.811–2.923)	1.389 (0.267–5.287)	0.021	0.35
Creatinine	1.623 (1.339–1.833)	1.52 (1.021–5.961)	0.171	
Cys	0.098 (0.065–0.123)	0.104 (0.051–0.173)	0.110	
Cysteine-glutathione disulphide	0.032 (0.016–0.042)	0.023 (0.01–0.048)	<0.001	0.84
**Cysteine-glutathione disulphide-Divalent**	**0.052 (0.03–0.085)**	**0.036 (0.014–0.065)**	**<0.001**	**1.44**
Cystine	1.128 (0.987–1.503)	1.25 (0.756–1.754)	0.068	
Decanoic acid	0.056 (0.035–0.125)	0.055 (0.022–0.175)	0.652	
Dihydroorotic acid	0.131 (0.085–0.145)	0.13 (0.085–0.165)	0.430	
gamma-Aminobutyric acid	0.051 (0.02–0.107)	0.045 (0.012–0.237)	0.332	
gamma-Butyrobetaine	0.126 (0.084–0.199)	0.092 (0.055–0.183)	<0.001	1.07
Gln	11.709 (9.18–15.218)	11.016 (7.37–14.963)	0.013	0.52
**Glu;threo-beta-methylaspartic acid**	**0.518 (0.376–0.857)**	**0.773 (0.222–2.049)**	**<0.001**	**0.72**
Gluconic acid	0.047 (0.041–0.059)	0.054 (0.032–0.263)	0.001	0.41
Glucose 6-phosphate; Fructose 6-phosphate; Glucose 1-phosphate	0.052 (0.027–0.091)	0.036 (0.012–0.113)	<0.001	0.71
Glucuronic acid; Galacturonic acid	0.032 (0.018–0.033)	0.032 (0.013–0.133)	0.035	0.54
Gly	2.866 (1.632–5.633)	2.054 (1.211–3.48)	<0.001	1.27
**Glyceric acid**	**0.064 (0.043–0.082)**	**0.078 (0.04–0.133)**	**<0.001**	**0.99**
**Glycerol-3-phosphate**	**0.011 (0.008–0.019)**	**0.018 (0.006–0.039)**	**<0.001**	**1.14**
Glycocholic acid	0.021 (0.006–0.115)	0.016 (0.005–0.115)	0.085	
Glycolic acid	0.066 (0.04–0.076)	0.062 (0.039–0.113)	0.386	
Glyoxylic acid	0.02 (0.014–0.026)	0.02 (0.013–0.032)	0.259	
Guanidinosuccinic acid	0.015 (0.009–0.023)	0.014 (0.008–0.089)	0.994	
Guanidoacetic acid	0.064 (0.037–0.097)	0.047 (0.03–0.11)	<0.001	0.76
Hippuric acid	0.035 (0.014–0.132)	0.029 (0.008–0.162)	0.045	0.18
**His**	**2.006 (1.81–2.576)**	**1.854 (1.172–2.888)**	**0.001**	**0.65**
Homoarginine or N6.N6.N6-Trimethyllysine	0.05 (0.031–0.068)	0.062 (0.032–0.097)	0.005	0.50
Homovanillic acid	0.014 (0.011–0.024)	0.017 (0.012–0.036)	<0.001	0.54
Hydroxyproline	0.174 (0.082–0.352)	0.186 (0.116–0.889)	0.404	
**Hypoxanthine**	**0.061 (0.016–0.111)**	**0.049 (0.022–0.166)**	**0.004**	**0.26**
Ile	2.74 (1.796–4.963)	2.731 (1.732–6.114)	0.392	
Indole-3-acetic acid	0.048 (0.033–0.073)	0.046 (0.029–0.145)	0.728	
Isethionic acid	0.02 (0.012–0.038)	0.019 (0.011–0.084)	0.185	
Isocitric acid	0.082 (0.057–0.109)	0.091 (0.055–0.189)	0.007	0.49
Kynurenine	0.051 (0.038–0.06)	0.047 (0.027–0.145)	0.388	
Lactic acid	5.526 (3.405–8.157)	6.551 (3.506–14.422)	0.004	0.54
Lauric acid	0.139 (0.064–0.203)	0.089 (0.045–0.46)	0.028	0.03
Leu	5.258 (3.7–8.267)	5.091 (2.734–9.618)	0.548	
Lys	3.802 (2.864–6.097)	4.281 (2.786–8.892)	0.169	
Malic acid	0.063 (0.034–0.096)	0.059 (0.034–0.13)	0.491	
**Met**	**0.36 (0.288–0.413)**	**0.318 (0.18–0.798)**	**0.016**	**0.04**
Methionine sulfoxide	0.078 (0.047–0.119)	0.088 (0.043–0.141)	0.152	
Mucic acid; Glucaric acid	0.029 (0.017–0.047)	0.034 (0.013–0.057)	0.214	
*N.N*-Dimethylglycine	0.082 (0.059–0.138)	0.108 (0.055–0.229)	<0.001	0.60
*N*5-Ethylglutamine	0.063 (0.044–0.124)	0.068 (0.04–0.919)	0.408	
*N*6.*N*6.*N*6-Trimethyllysine	0.047 (0.033–0.068)	0.047 (0.033–0.083)	0.388	
*N*-Acetyl-beta-alanine; *N*-Acetyl-beta-alanine	0.017 (0.011–0.028)	0.02 (0.01–0.034)	0.021	0.27
***N*** **-Acetyleucine**	**0.105 (0.098–0.163)**	**0.03 (0.008–0.265)**	**<0.001**	**0.67**
*N*-Acetylneuraminic acid	0.084 (0.068–0.128)	0.08 (0.047–0.14)	0.400	
O-Acetylcarnitine	1.069 (0.58–1.344)	0.824 (0.412–1.684)	<0.001	0.42
Octanoic acid	0.069 (0.051–0.143)	0.065 (0.039–0.155)	0.398	
Ornithine	1.299 (0.758–1.901)	1.194 (0.647–2.414)	0.392	
Pelargonic acid	0.07 (0.061–0.094)	0.08 (0.054–0.138)	0.007	0.66
**Phe**	**2.22 (1.992–2.857)**	**2.876 (1.511–9.056)**	**<0.001**	**0.72**
Pipecolic acid	0.047 (0.033–0.549)	0.059 (0.03–0.511)	0.131	
Pro	3.809 (1.937–8.386)	4.831 (2.252–9.739)	0.007	0.37
**Pyruvic acid**	**0.124 (0.085–0.273)**	**0.228 (0.078–0.418)**	**<0.001**	**1.58**
Quinic acid	0.038 (0.024–0.074)	0.036 (0.014–0.15)	0.101	0.39
Sarcosine	0.06 (0.046–0.119)	0.061 (0.03–0.186)	0.834	
SDMA	0.022 (0.018–0.028)	0.024 (0.016–0.084)	0.037	0.38
**Ser**	**1.836 (1.45–2.595)**	**1.524 (0.781–3.111)**	**<0.001**	**0.72**
S-Sulfocysteine	0.007 (0.006–0.011)	0.008 (0.005–0.014)	0.153	
Succinic acid; Methylmalonic acid	0.057 (0.035–0.074)	0.049 (0.027–0.073)	<0.001	0.63
Taurine	0.459 (0.342–2.117)	0.441 (0.265–3.031)	0.408	
Thr	2.163 (1.828–3.16)	2.19 (1.45–3.282)	0.652	
Threonic acid	0.214 (0.086–0.26)	0.23 (0.077–0.455)	<0.001	0.78
Trimethylamine *N*-oxide	0.033 (0.015–0.393)	0.053 (0.019–1.444)	0.091	
Trp	1.541 (1.236–1.879)	1.636 (0.921–2.904)	0.138	
**Tyr**	**1.711 (1.363–1.989)**	**1.977 (0.917–3.397)**	**0.001**	**0.65**
Urea	32.355 (21.09–47.464)	35.811 (21.836–70.115)	0.006	0.40
Uric acid	1.911 (1.381–2.553)	2.005 (1.237–3.465)	0.408	
Uridine	0.102 (0.086–0.168)	0.096 (0.074–0.212)	0.285	
Val	6.531 (5.305–11.255)	7.277 (4.631–12.402)	0.146	

Bold values indicate top ten metabolites in SHAP analysis.

**Table 2:** Evaluation of model results with comprehensive performance metrics for rheumatoid arthritis prediction.

Model	Metric	Value	BCI * (95%)
EBM	Accuracy	0.847	(0.776–0.918)
	F1-score	0.851	(0.781–0.922)
	Sensitivity	0.878	(0.752–0.954)
	Specificity	0.816	(0.68–0.912)
	Positive predictive value	0.827	(0.697–0.918)
	Negative predictive value	0.87	(0.737–0.951)
	AUC	0.901	(0.847–0.955)
	Brier score	0.129	(0.109–0.153)
LightGBM	Accuracy	0.806	(0.728–0.884)
	F1-score	0.812	(0.735–0.889)
	Sensitivity	0.837	(0.703–0.927)
	Specificity	0.776	(0.634–0.882)
	Positive predictive value	0.788	(0.653–0.889)
	Negative predictive value	0.826	(0.686–0.922)
	AUC	0.866	(0.806–0.926)
	Brier score	0.146	(0.133–0.185)
AdaBoost	Accuracy	0.776	(0.693–0.858)
	F1-score	0.784	(0.703–0.866)
	Sensitivity	0.816	(0.68–0.912)
	Specificity	0.735	(0.589–0.851)
	Positive predictive value	0.755	(0.617–0.862)
	Negative predictive value	0.8	(0.654–0.904)
	AUC	0.838	(0.775–0.902)
	Brier score	0.187	(0.172–0.208)

*: Bootstrapped confidence interval with 1000 repetitions.

## Data Availability

The raw data supporting the conclusions of this article will be made available by the authors on request.
